# Four bottlenecks restrict colonization and invasion by the pathogen *Ralstonia solanacearum* in resistant tomato

**DOI:** 10.1093/jxb/erz562

**Published:** 2019-12-24

**Authors:** Marc Planas-Marquès, Jonathan P Kressin, Anurag Kashyap, Dilip R Panthee, Frank J Louws, Nuria S Coll, Marc Valls

**Affiliations:** 1 Centre for Research in Agricultural Genomics (CRAG), CSIC-IRTA-UAB-UB, Campus UAB, Bellaterra, Barcelona, Spain; 2 Department of Entomology and Plant Pathology, North Carolina State University, Raleigh, NC, USA; 3 Department of Horticultural Science, North Carolina State University, Mountain Horticultural Crops Research and Extension Center, Mills River, NC, USA; 4 Department of Horticultural Science, North Carolina State University, Raleigh, NC, USA; 5 Department of Genetics, University of Barcelona, Barcelona, Spain; 6 Swedish University of Agricultural Sciences, Sweden

**Keywords:** Bacterial wilt, disease resistance, *Ralstonia solanacearum*, tomato, vascular pathogen, xylem

## Abstract

*Ralstonia solanacearum* is a bacterial vascular pathogen causing devastating bacterial wilt. In the field, resistance against this pathogen is quantitative and is available for breeders only in tomato and eggplant. To understand the basis of resistance to *R. solanacearum* in tomato, we investigated the spatio-temporal dynamics of bacterial colonization using non-invasive live monitoring techniques coupled to grafting of susceptible and resistant varieties. We found four ‘bottlenecks’ that limit the bacterium in resistant tomato: root colonization, vertical movement from roots to shoots, circular vascular bundle invasion, and radial apoplastic spread in the cortex. Radial invasion of cortical extracellular spaces occurred mostly at late disease stages but was observed throughout plant infection. This study shows that resistance is expressed in both root and shoot tissues, and highlights the importance of structural constraints to bacterial spread as a resistance mechanism. It also shows that *R. solanacearum* is not only a vascular pathogen but spreads out of the xylem, occupying the plant apoplast niche. Our work will help elucidate the complex genetic determinants of resistance, setting the foundations to decipher the molecular mechanisms that limit pathogen colonization, which may provide new precision tools to fight bacterial wilt in the field.

## Teaser

The spatio-temporal dynamics of the tomato–*Ralstonia solanacearum* interaction reveal spread of the pathogen out of the xylem and four different limitations to bacterial colonization in resistant tomato.

## Introduction

Bacterial wilt caused by the *Ralstonia solanacearum* species complex is a disease of major economic importance, impacting the production of solanaceous crops, legumes, banana, ginger, and ornamentals (Hayward, 1994). *R. solanacearum* enters the roots through wounds, colonizes the xylem tissue, moves up into the stem, and causes a rapid, permanent wilt through a combination of high bacterial density and mass production of extracellular polysaccharides (Hayward, 1991; Grimault and Prior, 1993; McGarvey *et al.*, 1999; Schell, 2000). *R. solanacearum* can move across the root following either an apoplastic pathway through the middle lamella or a pseudo-symplastic pathway via the xylem vessel lumens and axillary pits (Schell, 2000).

The management of bacterial wilt is challenging due to the aggressiveness of *R. solanacearum* and its broad geographical distribution, wide host range, and persistence in soil and water for long periods (Genin, 2010; Mansfield *et al.*, 2012). Strong quantitative resistance to bacterial wilt in tomato has been available for many decades, but has been successfully deployed only in small-fruited varieties (<200 g) and rootstocks for grafting, due to a seemingly unbreakable linkage between small fruit size and resistance (Scott *et al.*, 2005; Rivard and Louws, 2008). The Hawaii breeding line series, particularly Hawaii 7996, is the most effective source of resistance against various strains of *R. solanacearum* under different environmental conditions, and are widely used rootstocks for management of bacterial wilt (Grimault *et al.*, 1994*a*; Prior *et al.*, 1996; Wang *et al.*, 1998). The commercially successful hybrid Shield has been the most commonly planted rootstock for bacterial wilt resistance in North Carolina, USA, in recent years; in this location, this hybrid is highly resistant in fields with moderate disease pressure (Suchoff *et al.*, 2015) but shows an intermediate resistance under strong disease pressure (Kressin, 2018). Resistance in a mapping population derived from Hawaii 7996 (resistant) × West Virginia 700 (susceptible) has been reported to be mainly quantitative, involving two major quantitative trait loci (QTLs) located in chromosomes 12 and 6 (Bwr-12 and Bwr-6), accounting for 18–56% and 11–22% of the phenotypic variation, respectively (Wang *et al.*, 2013), and three minor loci (Bwr-3, Bwr-4, and Bwr-8). Some of these QTLs are strain- and/or environment-specific (Thoquet *et al*., 1996*a*, *b*; Mangin *et al.*, 1999; Wang *et al.*, 2000; Carmeille *et al.*, 2006; Wang *et al.*, 2013).

Initial studies of *R. solanacearum* colonization in several resistant and susceptible tomato varieties reported that bacterial wilt resistance was associated with the capability of the plant to limit bacterial spread from the root collar to the midstem, and not with limited root invasion (Grimault and Prior, 1993; Nakaho, 1997*a*). However, when similar experiments were performed without wounding the roots, limited bacterial growth in Hawaii 7996 was observed in all tissues analyzed (taproot, hypocotyl, petiole and mid-stem; McGarvey *et al.*, 1999).

Studies analyzing plant colonization in grafted tomatoes showed that the bacterium was capable of crossing the graft junction into the susceptible scion. Hawaii 7996 rootstocks were the most efficient in limiting susceptible scion infection to 38% and wilting to only 10% in conditions where susceptible varieties were 100% infected and wilted (Nakaho *et al.*, 2004).

Microscopic studies of bacterial wilt in tomato described the presence of inducible physico-chemical barriers (tyloses, gums, and modifications to the primary cell wall) that seemed to limit bacterial spread in the resistant variety Caraïbo (Grimault *et al.*, 1994*b*). Light microscopic examination of upper hypocotyls revealed that bacterial masses were present only in the primary xylem tissues of resistant LS-89 plants (derived from the line Hawaii 7998), whereas bacteria were found in both the primary and secondary xylem tissues of the susceptible line Ponderosa (Nakaho, 1997*a*). Thus, disease severity in *R. solanacearum*-infected tomato plants was proposed to correlate with the extent of bacterial invasion into the secondary xylem tissues (Nakaho, 1997*a*, *b*). This limitation of pathogen movement from the protoxylem or the primary xylem to other xylem tissues was most conspicuous in Hawaii 7996 (Nakaho *et al.*, 2004). Other studies reported that the cell walls were thicker in parenchyma and vessel cells of infected xylem tissues in resistant LS-89 than in susceptible Ponderosa or mock-inoculated plants (Nakaho *et al.*, 2000). Accumulations of electron-dense materials in vessels and parenchyma cells were also more apparent in LS-89, while Ponderosa showed necrosis in all parenchyma cells adjacent to vessels containing bacteria (Nakaho *et al.*, 2000). A recent microscopic study of the distribution of *R. solanacearum* in roots of Hawaii 7996 and the susceptible cultivar West Virginia 700 found that colonization of the root vascular cylinder was delayed and movement inside the vasculature was spatially restricted in Hawaii 7996 (Caldwell *et al.*, 2017). Together, these studies underscore the existence of a complex set of events that restrict bacterial colonization in space and time in resistant varieties of tomato. However, a systematic investigation of *R. solanacearum* invasion patterns at the whole-plant and tissue level is lacking.

Here, we applied luminescent and fluorescent bacteria for the characterization of resistance to bacterial wilt in tomato root, hypocotyl, and stem organs at the tissue level. We compared highly susceptible, moderately resistant, and highly resistant grafted tomato plants using a standard soil-based seedling grafting method and an *in vitro* grafting method. We propose an integrative model for bacterial wilt in resistant tomato lines that highlights the importance of four different ‘bottlenecks’ that limit bacterial colonization: (i) invasion of the root; (ii) vertical movement upwards to the stem; (iii) circular passage from vessel to vessel; and (iv) escape from the xylem and radial spread into the pith/cortex tissues.

## Materials and methods

### Plant and bacterial materials and growth conditions

The tomato (*Solanum lycopersicum*) lines used in this study were the highly susceptible commercial variety Marmande (Leroy Merlin), the moderately resistant commercial hybrid rootstock Shield (Rijk Zwaan), and the highly resistant public open-pollinated breeding line Hawaii 7996.

For *in vitro* experiments, tomato seeds were surface sterilized in 35% bleach and 0.02% Triton-X 100 for 10 minutes and then rinsed with sterile distilled water five times before sowing them on semi-solid medium [Murashige and Skoog (MS) with agar] in square culture plates (Sudelab S.L.). Plates were placed standing upright in a walk-in tissue culture growth chamber set at 22 °C under long-day light conditions.

For pot experiments, plants were grown on soil (Substrate 2, Klasmann-Deilmann GmbH) mixed with perlite and vermiculite (30:1:1) in a growth chamber: either a FITOCLIMA 1200 (Aralab) set at 27 °C, with LED lighting, or a SCLAB S.L. set at 25 °C, with fluorescence lighting. In both cases, conditions were set at 60% humidity and 12 h day/night with light intensity of 120–150 µmol·m^–2^·s^–1^.

All assays were performed using *R. solanacearum* GMI1000 strain. The constructs PpsbA::LuxCDABE and PpsbA::GFPuv generated by Cruz *et al.* (2014) were naturally transformed into *R. solanacearum* GMI1000 to generate the luminescent and fluorescent reporter strains, respectively. *R. solanacearum* was grown as previously described (Planas-Marquès *et al.*, 2018).

### Plant grafting

For *in vitro* grafting, seeds were sown on to sterile filter paper placed on MS-containing plates. Eight days after germination (7 for Marmande to obtain equivalent stem diameters), the cotyledons were removed and the plants were cut at a perpendicular angle 1–2 cm below the cotyledons using sterile tools. For double-grafted plants, two cuts were made 2–3 cm apart. Rootstocks and scions were transferred to fresh plates without filter paper and matched with the corresponding reciprocal tissues without any stabilizing device. Plates were kept standing upright in the growth chamber. After 10 days, successfully healed plants were either pin-inoculated with the luminescent strain of *R. solanacearum* and monitored over time or transferred to soil-containing pots and grown as described for the pathogenicity assays after acclimation for 48 h in transparent boxes (Altuna 2594005, Stewart Garden) with the vented lids opened after 24 h.

For standard grafting, plants with stems 1.5–2 mm in diameter (9 days after sowing) were grafted 2 cm below the cotyledons using a 70° angle cut and 1.6 or 2 mm diameter grafting clips (Bato Plastics B.V.). Grafted plants were kept in misted acclimation boxes in growth chambers and acclimated to light (24 h darkness, 24 h at 10% light, 24 h at 50% light) and then to ambient humidity (by opening the vents 4 days after grafting and partly opening the lid for 48 h before removing it).

### Plant inoculation and pathogenicity assays

For *in vitro* assays, 10-day-old plantlets or plantlets 10 days after grafting were pin-inoculated 1 cm below the root collar using a sterile 0.3×13 mm needle (30G×½″, BD Microlance, Becton Dickinson) submerged in a 10^6^ colony-forming units (CFU)·ml^–1^ (OD_600_=0.001) *R. solanacearum* suspension. Plates were kept in a growth chamber (25 °C day, 22 °C night), and wilting symptoms were recorded and bacterial invasion visualized as described below.

For soil-drenching inoculations, plants were grown until they reached the 7–9 true leaf stage (4–5 weeks after sowing, and 5–6 weeks for grafted plants). Inoculations were performed by pouring 40 ml of a 10^7^ CFU·ml ^–1^ (OD_600_=0.01) bacterial suspension on every pot after making four holes in the soil with a disposable 1 ml pipette tip. Infected plants were kept in a growth chamber set at 27 °C and scored for wilting symptoms using a scale from 0 to 4: 0=healthy plant with no wilt, 1=25%, 2=50%, 3=75%, and 4=100% of the canopy wilted. To assess shoot colonization, 4–5-week-old plants were pin-inoculated with 10 µl of a 10^6^ CFU·ml^–1^ suspension (Ishihara *et al.*, 2012) when indicated (see Supplementary Fig. S9 at *JXB* online).

### Assessment of bacterial invasion

Invasion of tomato tissues by *R. solanacearum* was assessed using the luminescent and fluorescent strains. For *in vitro* assays, pin-inoculated plants were photographed using a live imaging system (ChemiDoc Touch Imaging System, Bio-Rad) as previously described by Cruz *et al.* (2014), using a 5-minute exposure time with 3×3 sensitivity. Images were processed using Image Lab software (Bio-Rad). Inoculated soil-grown plants were uprooted and the roots were surface sterilized in water with ~5–10% bleach for at least 1 minute followed by a wash in water. Plants inoculated with the luminescent strain were sliced from the apex to the roots using a sterile razor blade. Transverse sections 1 mm thick and the two halves of longitudinal slices 1–2 cm in length were placed flat on a square plate with a misted lid and visualized using a live imaging system as described above. For each location, a 0.5 cm section was excised and incubated for at least 30 minutes in a sterile 2 ml tube with 200 µl of sterile distilled water. Luminescence was measured with a luminometer (FB 12, Berthold Detection Systems). The relative light units per second (RLU·s^–1^) were related to CFU·g^–1^ tissue after dilution plating of samples and CFU counting 24 h later.

Plants inoculated with the fluorescent strain were dissected as described above and photographed using binocular microscopy with a UV fluorescent lamp (BP330-385 BA420 filter) and a SZX16 stereomicroscope equipped with a DP71 camera system (Olympus). Quantification of mean fluorescence in the green, blue, and red channels was achieved using ImageJ software.

### Statistical analysis

Statistical analyses were performed using Statgraphics software. All statistical tests are indicated in the respective figure legends.

## Results

### The first ‘bottleneck’ in *R. solanacearum* colonization: the root-to-shoot boundary

**Fig. 1.**
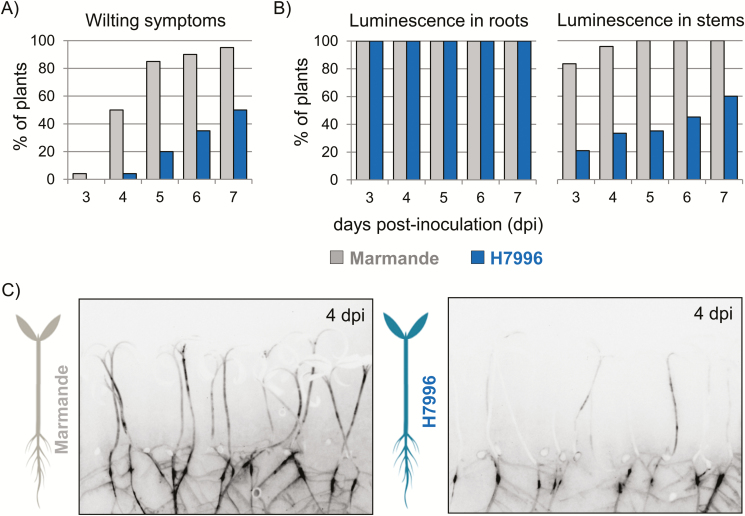
Evaluation of *R. solanacearum* colonization in *in vitro-*grown resistant and susceptible tomato plants. Tomato seedlings of the susceptible variety Marmande and the resistant variety Hawaii 7996 (H7996) were pin-inoculated in the roots with a luminescent *R. solanacearum* strain, and bacterial colonization and wilting symptoms were evaluated over time. (A) Percentage of plants showing wilting symptoms. (B) Percentage of plants colonized in the roots and stems based on the luminescence signal emitted by the reporter strain. (C) Representative photographs showing infected seedlings at 4 days post-inoculation (dpi). The outline of the plants is due to background light from photosynthetic tissues, while luminescence is shown as darker areas. Saturation was never reached. The experiment was repeated three times with similar colonization kinetics; *n*=20 plants per variety. (This figure is available in colour at *JXB* online.)

Limited shoot colonization by *R. solanacearum* in resistant tomatoes has been proposed to be due to reduced spread of the bacterium from the root to the stem (Grimault and Prior, 1993; Nakaho, 1997*a*) and/or limited root invasion in resistant varieties (McGarvey *et al.*, 1999; Caldwell *et al.*, 2017). To clearly define the location within the plant at which resistance was acting, we made use of a constitutively luminescent *R. solanacearum* that we had previously generated (Cruz *et al.*, 2014) to follow bacterial colonization in resistant and susceptible tomato plants in a non-disruptive manner. For this, we established a miniaturized *in vitro* tomato–*R. solanacearum* infection system. Tomato seedlings were grown on semi-solid medium and pin-inoculated in the roots with the luminescent strain. This forced inoculation ensured the infection of all plants to enable the study of bacterial spread in the plant tissues. Disease symptoms were recorded as the percentage of wilted plants (Fig. 1A) and plants were photographed using a live imaging system over time. This non-destructive assay mimicked the disease symptomatology observed in field or greenhouse conditions under strong disease pressure, as indicated by the reduced wilting of the resistant line Hawaii 7996 (H7996) compared with the susceptible line Marmande (Fig. 1A). While all tomato roots in both lines were colonized 3 days post-inoculation (dpi), shoot colonization was clearly delayed and reduced in H7996 compared with Marmande (Fig. 1B). A representative photograph of the assay at 4 dpi, when the susceptible plants start to wilt, is presented in Fig. 1C. This image shows that, besides the difference in shoot colonization in the two varieties, a colonization ‘bottleneck’ exists in resistant plants at the root collar. In addition, luminescence intensity was lower in the roots of H7996 (Fig. 1C), indicating lower bacterial loads compared with Marmande.

### Resistant rootstocks reduce plant invasion and limit bacterial multiplication in the roots of grafted plants

**Fig. 2.**
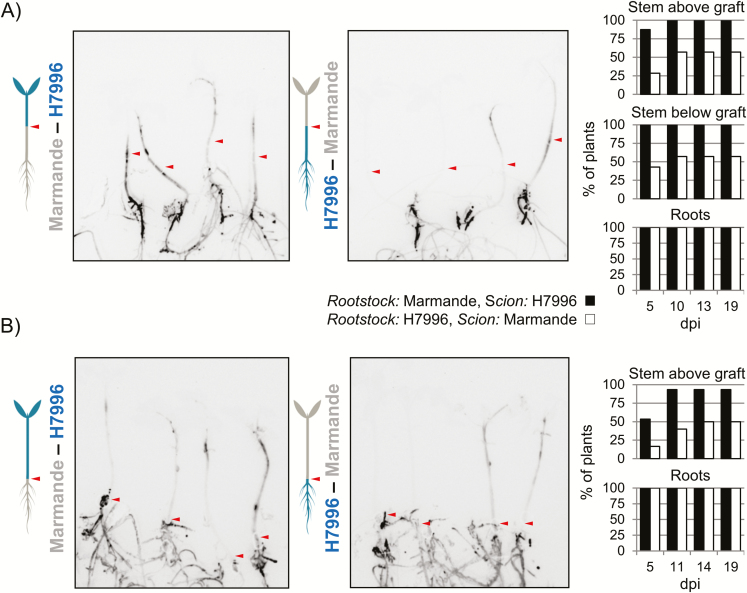
Bacterial shoot colonization in Marmande and Hawaii 7996 (H7996) grafted plants. Seedlings of Marmande and H7996 were grafted at the mid-stem (A) or the root collar (B) and then pin-inoculated in the roots with the luminescent *R. solanacearum* strain. A representative photograph of reciprocally grafted plants is shown for each graft type at 10 dpi. The percentages of plants colonized in the roots and the tissues immediately below and above the graft are shown on the right. Arrowheads indicate the grafting junction. Both experiments were repeated at least three times, with similar colonization kinetics. In (A), *n*=7–8 plants per grafting combination; in (B), *n*=12–15 plants per grafting combination. (This figure is available in colour at *JXB* online.)

To analyze the contribution of the roots to resistance in further detail, we grafted rootstocks and scions of Marmande and H7996. Grafts were made at the upper hypocotyl and at the root collar, and bacterial colonization and disease progression were evaluated using the luminescent *R. solanacearum* strain after pin-inoculation of the roots (Fig. 2). Resistant H7996 rootstocks hampered bacterial colonization of Marmande scions, while Marmande roots did not prevent colonization of H7996 scions (Fig. 2A). The presence of a resistant root system was sufficient to cause a reduction in shoot colonization, as stem luminescence was comparable in grafted plants with or without a resistant lower stem (Fig. 2B).

To strengthen these observations, we investigated root colonization by *R. solanacearum* in fully developed plants inoculated by soil drenching with the luminescent *R. solanacearum* strain. The tomato variety Shield, which is moderately resistant to bacterial wilt, was introduced in these experiments for comparison with the susceptible Marmande and highly resistant H7996 varieties. We imaged whole roots of plants of each variety obtained at 6 dpi (see Supplementary Fig. S1 at *JXB* online), when plants were already showing wilting symptoms (Supplementary Fig. S2). Roots of Marmande displayed strong luminescence intensity, while roots of Shield or H7996 displayed weak luminescence (Supplementary Fig. S1A). This phenomenon was consistent regardless of the intensity of the signal in the stem or the level of wilting, and correlated with our previous results obtained using the miniaturized *in vitro* system (Fig. 1).

To quantify the reduced root colonization with *R. solanacearum* in resistant varieties, we measured bacterial loads in the taproot at 3 dpi, when susceptible plants started to show symptoms. Bacterial concentrations were calculated from luminescence units (RLU) measured from taproots with a luminometer, based on the extremely high correlation (*r*^2^=0.96, *P*<0.0001) between the luminescence emitted by the tissue samples and the bacterial CFU (Supplementary Fig. S3). This experiment revealed that the resistant rootstocks Shield and H7996 had a significantly lower mean bacterial density at the root compared with the susceptible variety Marmande, which exhibited bacterial concentrations two orders of magnitude higher (Supplementary Fig. S1B).

### The second ‘bottleneck’: resistant shoots restrict vertical movement of bacteria along the xylem

**Fig. 3.**
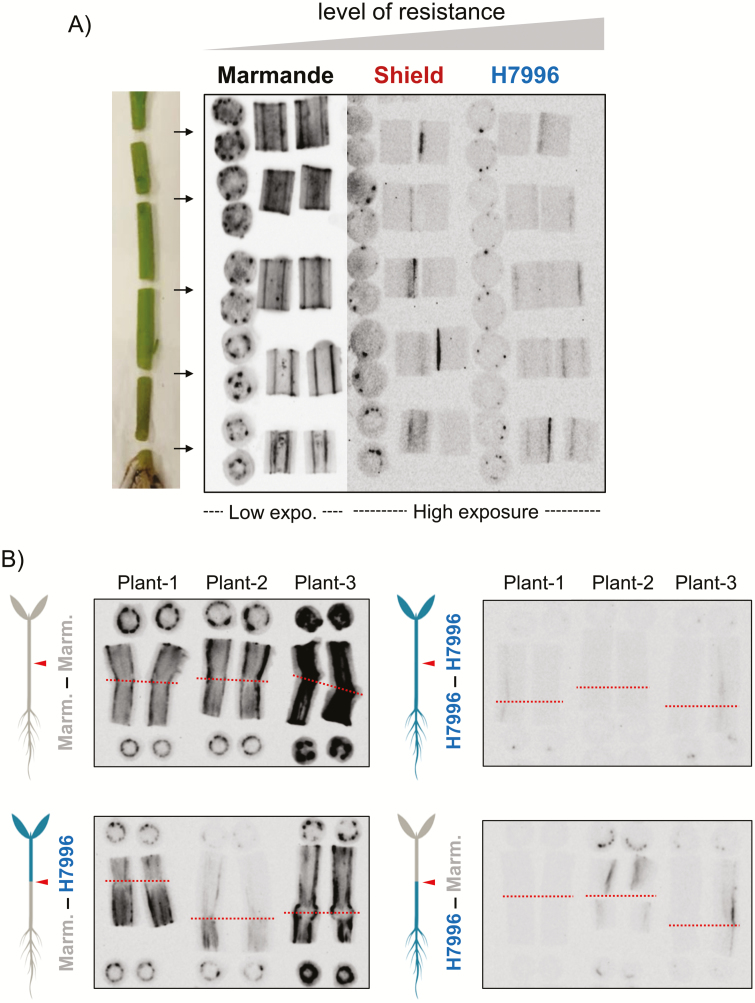
Vertical movement of *R. solanacearum* in tomato shoots. Tomato plants (4–6 weeks old) of (A) the susceptible variety Marmande, the moderately resistant variety Shield, and the highly resistant variety H7996, and (B) reciprocally grafted Marmande and H7996 plants grown in pots were soil inoculated with luminescent *R. solanacearum*. Shoot sections were obtained at 6 dpi and photographed in a live imager. In (A), photographs show each bisected fragment and its top and bottom slices. Sections were obtained at the base of the hypocotyl, the distal hypocotyl (immediately below the cotyledons), and internodes 1, 2, and 3. In Image Lab software (Bio-Rad) the following ‘High’/‘Low’/‘Gamma’ values were used for low and high exposure settings, respectively: 10000/60/1 and 1300/60/2. In (B), sections were obtained above and below the graft junction. Arrowheads and dotted lines indicate the position of the graft junction. (This figure is available in colour at *JXB* online.)

Next, we investigated *R. solanacearum* shoot colonization in soil-inoculated fully developed Marmande, Shield, and H7996 plants. Intact 4–5-week-old plants grown in pots could not be imaged for luminescence because of size limitations and reduced sensitivity resulting from the stem thickness. Therefore, we obtained 1–2 cm stem sections up to the third internode from plants 6 dpi, when wilting symptoms could be observed (Supplementary Fig. S2). In order to track the movement of luminescent bacteria throughout the stem, slices were taken from the top and bottom of each section and the remaining stem was divided longitudinally into two pieces. Representative photographs of all sections from a plant of each variety are presented in Fig. 3. In all cases, the luminescence matched the location of xylem bundles, indicating that bacteria were mostly confined to the xylem at this stage of infection. As expected, bacterial colonization in the shoot was much more apparent in Marmande, as indicated by the intense luminescence observed, compared with the resistant varieties, in which luminescence was in most cases detected only at a higher exposure setting (Fig. 3A Supplementary Fig. S4). In addition, the number of luminescent bundles decreased occasionally with height in the resistant varieties, whereas it remained constant in the susceptible Marmande plants. In summary, resistant tomato lines display a lower number of colonized xylem fiber bundles and some limited bacterial vertical movement along the vessels, compared with susceptible plants (Fig. 3).

To avoid between-plant variation in colonization and directly compare the behavior of susceptible and resistant tissues when confronted with equivalent bacterial loads, we characterized *R. solanacearum* distribution in reciprocally grafted plants. We used adult plants inoculated by soil drenching and monitored the vertical movement of the luminescent bacterial strain in the hypocotyl region (where grafting was performed) at 6 dpi (Fig. 3B). Colonized vessels and luminescence were almost undetectable in self-grafted resistant H7996 (Fig. 3B), similar to what was observed in non-grafted plants (Fig. 3A). Self-grafting of the Marmande variety demonstrated that grafting *per se* did not restrict vertical movement (Fig. 3B). Colonization was hampered in H7996 scions grafted on to Marmande rootstocks, and was higher in Marmande scions than in the H7996 rootstocks grafted on to them (Fig. 3B). These results demonstrated that at comparable bacterial concentrations, vertical colonization is inhibited and overall bacterial density is strongly reduced along the xylem of H7996 compared with Marmande. Similar results were observed in Marmande–Shield grafting combinations (Supplementary Fig. S5).

**Fig. 4.**
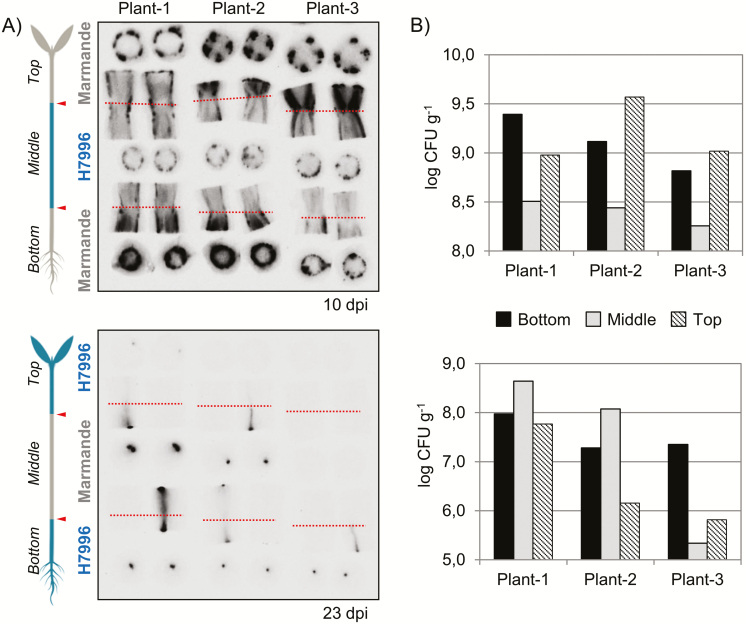
Bacterial shoot colonization in Marmande and H7996 double-grafted plants. Seedlings of Marmande and H7996 were double-grafted in the middle of the stem, transferred to pots, and grown for 3–4 weeks. They were then inoculated with the luminescent *R. solanacearum* strain by soil drenching. (A) Shoot sections from the hypocotyl were obtained at 10 dpi (upper panel) or 23 dpi (lower panel) and photographed in a live imager. ‘Bottom’ and ‘Top’ refer to the basal and distal hypocotyl locations, respectively; ‘Middle’ refers to the region between the two graft junctions. Graft junctions are indicated by arrowheads and dotted lines. (B) Bacterial loads in the shoots of the plants shown in (A) were calculated from the luminescence and are expressed as log CFU g^–1^ tissue. (This figure is available in colour at *JXB* online.)

A decrease in vertical colonization could be explained by a timing artefact: if luminescence photographs were taken too soon for the bacteria to grow in the resistant scion, this would give a false impression of hampered invasion. To rule out this possibility, we exchanged a fragment of hypocotyl between Marmande and H7996 plants in a double-grafting approach (Supplementary Fig. S6). Grafted plants contained a 2 cm fragment of the hypocotyl from one of the varieties in-between the basal and distal hypocotyl regions of the other variety (Supplementary Fig. S6A, B). The double-grafted plants were grown on soil to the 7–9 true leaf stage and were infected with the luminescent *R. solanacearum* strain (Fig. S6C–G). As expected, plants that contained the roots and basal hypocotyl from Marmande wilted to a similar extent to plants with Marmande rootstocks (Supplementary Figs S2, S6D, E). We observed and quantified bacterial movement along the xylem in the two combinations of double-grafted plants using luminescence (Fig. 4). Marmande rootstocks were heavily colonized by *R. solanacearum*, and bacterial density decreased as soon as the pathogen crossed the first grafting junction and encountered H7996 tissue. When *R. solanacearum* moved upwards into susceptible tissue for the second time, it again multiplied to high concentrations (Fig. 4). The opposite result was observed in the reciprocal grafting: colonization with *R. solanacearum* was hampered in H7996 rootstocks, especially at 10 dpi (Supplementary Fig. S6F, G), increased in Marmande hypocotyls, and decreased when the bacteria crossed the second grafting junction and encountered H7996 tissue again (Fig. 4). Altogether, these results demonstrate the ability of H7996 to restrict vertical movement of *R. solanacearum* along the xylem in a root-independent manner.

### Plant wilting is determined by a bacterial density threshold in the hypocotyl

**Fig. 5.**
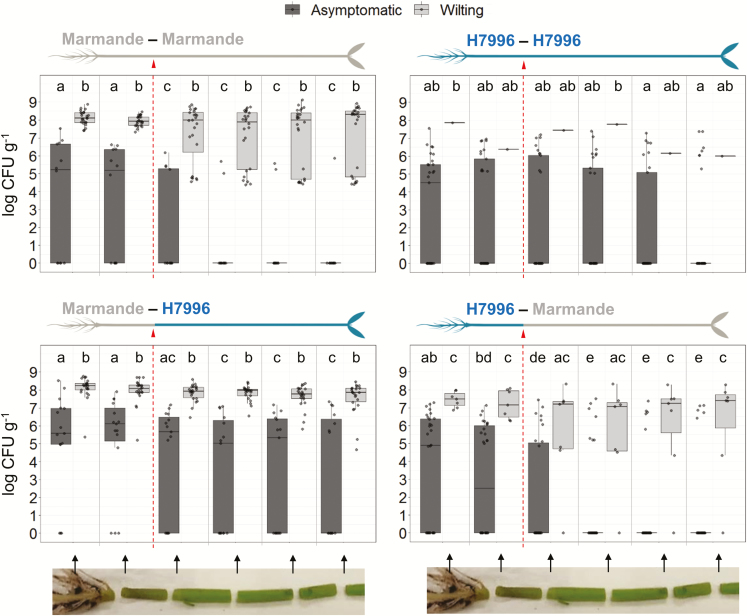
Density of colonization of *R. solanacearum* assessed over the height of grafted tomato plants. Bacterial concentrations at different heights in the tissues of wilting (light grey) and asymptomatic (dark grey) grafted plants are shown. Luminescence was measured with a luminometer in 0.5 cm sections from at least 30 inoculated plants per grafting combination. Bacterial counts were calculated from the luminescence and are expressed as log CFU g^–1^ tissue. Each dot represents one plant. Only one self-grafted H7996 plant wilted, hence the lack of a boxplot. Values between 0 and 4 lie below the threshold for luminescence detection (see Supplementary Fig. S3) and are considered as 0 here. From left to right, sections correspond to: taproot, basal hypocotyl, distal hypocotyl, and internodes 1, 2, and 3. The dashed line and arrowhead indicate the location of the graft junction. Different letters above each boxplot indicate statistically significant differences (α=0.05, Fisher’s least significant difference test). In each boxplot, the whiskers extend vertically from the hinges (the upper and lower bounds of the box) to the largest (upper whisker) or smallest (lower whisker) value no further than 1.5 × the interquartile range (the distance between the first and third quartiles) from the hinge. Dots beyond the ends of the whiskers are outliers. The horizontal band inside each box indicates the median. (This figure is available in colour at *JXB* online.)

To trace the vertical movement of bacteria in the plant in a quantitative manner, we measured bacterial loads from the taproot to the third internode in at least 30 plants per grafting combination, sampled at different times (3–10 dpi), which showed a range of wilting symptoms. The results in Fig. 5 clearly show that, regardless of the degree of susceptibility, asymptomatic tomato plants contained bacterial concentrations generally lower than 10^7^ CFU g^–1^ tissue and wilted plants always showed bacterial counts above this threshold in the taproot and basal hypocotyl, although they may have had lower numbers of bacteria in the shoot above the cotyledons. Additionally, the hypocotyl seemed to act as an additional vertical threshold in susceptible plants, since asymptomatic Marmande plants were often colonized below the hypocotyl but the plants always wilted when the bacteria moved above the hypocotyl (Fig. 5). In contrast, when H7996 scions were grafted on to Marmande rootstocks —a situation in which the barrier in the roots of the resistant variety is absent— the tissues of the resistant variety were able to cope with high bacterial concentrations in the shoot and the plants remained asymptomatic (Fig. 5). Similar results were observed using the Shield variety (Supplementary Fig. S7).

### The third and fourth ‘bottlenecks’: resistant shoots restrict circular and radial movement of bacteria

**Fig. 6.**
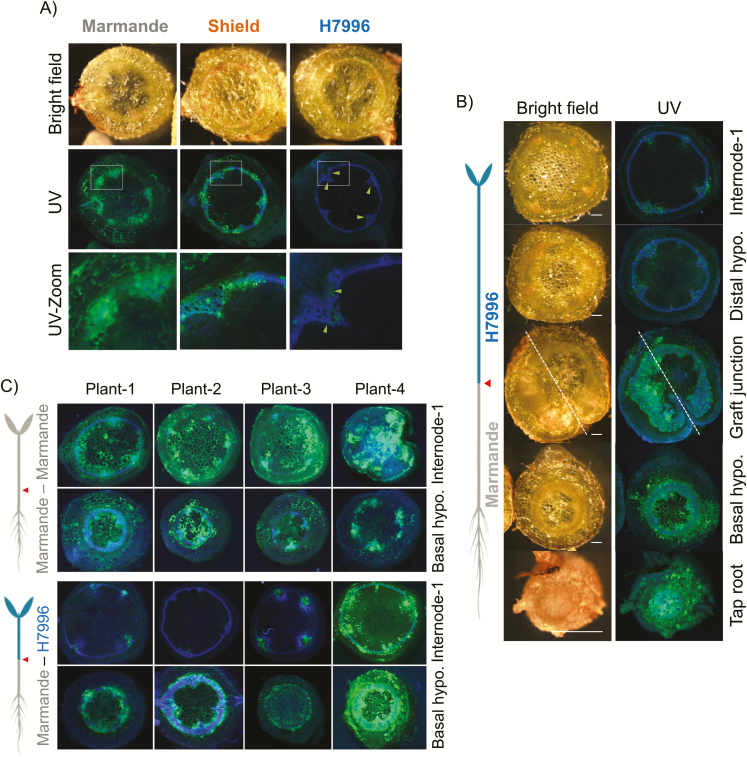
Distribution of a fluorescent *R. solanacearum* strain in susceptible and resistant tomato shoots. (A) Plants (4–5 weeks old) of the susceptible variety Marmande, the moderately resistant variety Shield, and the highly resistant variety H7996 grown in pots were inoculated with a fluorescent *R. solanacearum* strain by soil drenching. Basal hypocotyl stem sections were obtained and photographed with a fluorescence stereomicroscope under white light (upper panels) and UV light (middle and lower panels). The lower panels show a magnification of the areas indicated with rectangles in the middle panels. The sections were visualized through a UV light filter, highlighting the autofluorescence of lignin in blue and the fluorescence emitted by the bacteria in green. Green dots correspond to clumps of bacteria. Arrowheads indicate xylem vessels with limited colonization. (B) Plants consisting of H7996 scions grafted on to Marmande rootstocks were grown and inoculated with the fluorescent strain, and transverse sections taken at different heights below and above the graft junction were photographed with a fluorescence stereomicroscope. (C) Fluorescence photomicrographs of highly colonized and fully wilted Marmande and H7996 shoots from four plants at the basal hypocotyl and the first internode.

In order to examine the patterns of colonization within the stems at the tissue level, we inoculated 4-week-old Marmande, H7996, and Shield plants grown in pots with a *R. solanacearum* strain constitutively expressing GFPuv (Cruz *et al.* 2014) and observed slices of the shoots with a fluorescence stereomicroscope. Fig. 6A shows representative images of transverse hypocotyl sections of the three tomato varieties at 8 dpi. At this stage, the stem xylem tissue was arranged into four primary bundles and typically two to four smaller secondary bundles, connected by the interfascicular cambium formed by xylem parenchyma and some xylary fibers. The microscopic images indicate that *R. solanacearum* can move horizontally from vessel to vessel (circular movement) and from the vessels to the adjacent parenchyma tissues (radial movement). In the susceptible variety Marmande, fluorescent bacteria occupied almost the entire vascular ring and even extended radially to the apoplast of the pith and cortical tissues (Fig. 6A). In contrast, in the resistant variety H7996 bacteria were confined to a few single xylem vessels (Fig. 6A). The moderately resistant variety Shield showed an intermediate phenotype, with colonization more restricted to the vascular ring and limited radial spread to neighboring tissues (Fig. 6A).

The extremely limited vertical colonization of the xylem in H7996 hampered precise characterization of the circular and radial bacterial movements in the shoots. To overcome this limitation, we grafted H7996 scions on to Marmande rootstocks, to enable high bacterial numbers to reach the resistant stem tissues (Figs 3B, 4, 5), and inoculated the grafted plants with the fluorescent *R. solanacearum* strain by soil drenching. Examination of shoot sections obtained at different shoot heights at 8 dpi revealed extensive vertical, circular, and radial colonization of the Marmande tissues below the graft (Fig. 6B, Supplementary Fig. S8). The section at the graft junction showed that the H7996 tissues immediately blocked the spread of the bacterium both circularly through the xylem ring and radially to the pith and cortical tissues (Fig. 6B). These restrictions became more apparent in higher sections consisting exclusively of resistant tissue (Fig. 6B, Supplementary Fig. S8).

To better compare the behavior of *R. solanacearum* in resistant and susceptible tissues, we repeated this experiment using a larger number of plants, and observed shoot sections of resistant scions that showed the most pronounced wilting with the fluorescence stereomicroscope. Fig. 6C shows these H7996 shoot sections confronted with a high bacterial inoculum introduced from the susceptible rootstock, compared with Marmande shoot sections. Notably, radial movement of bacteria from the highly colonized xylem bundles was strongly restricted in the H7996 shoots, even when the xylem tissue was highly colonized (Fig. 6C right panel). This restriction was also observed when the fluorescent *R. solanacearum* strain was directly pin-inoculated into the shoots (Supplementary Fig. S9).

**Fig. 7.**
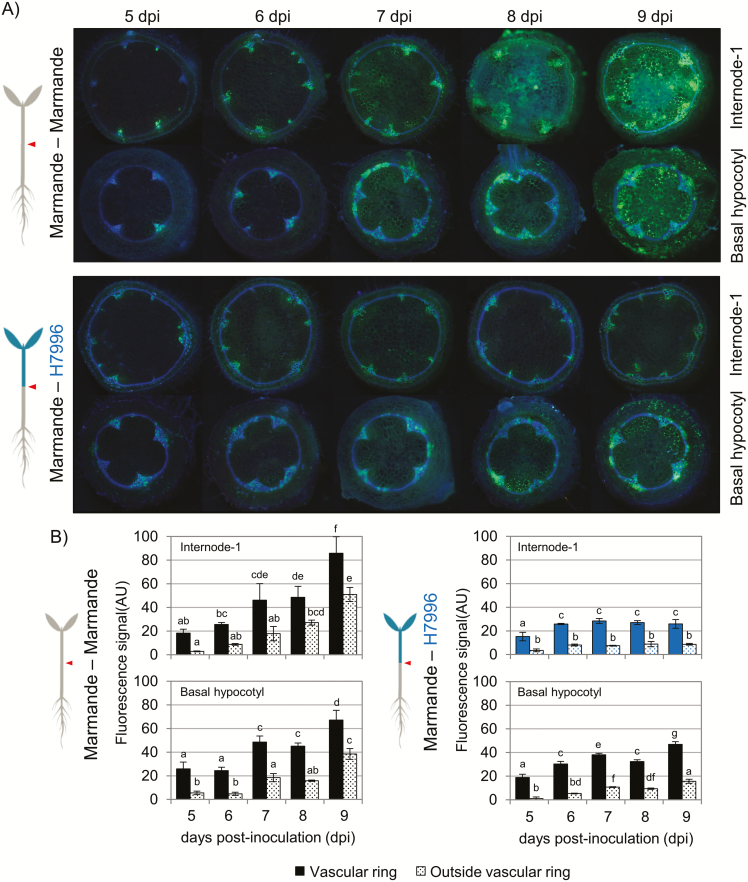
Time course of invasion of the fluorescent *R. solanacearum* strain in grafted tomato shoots. (A) Fluorescence photomicrographs of self-grafted Marmande and plants consisting of H7996 scions grafted on to Marmande rootstocks inoculated with the fluorescent *R. solanacearum* strain. Sections were taken at the basal hypocotyl and the first internode. The sections were visualized through a UV light filter, highlighting the autofluorescence of lignin in blue and the fluorescence emitted by the bacteria in green. (B) Quantification of fluorescence signal (AU, arbitrary units) within and outside the vascular ring in the basal hypocotyl and the first internode of plants from each stage of the infection shown in (A). Three biological replicates (*n*=3) were used. Data presented are means ± SE. Different letters above the bars indicate statistically significant differences (α=0.05, Fisher’s least significant difference test).

Finally, we performed a time-course invasion assay in which we quantified the amounts of bacteria that were moving outside the vascular ring over time (Fig. 7). We observed that *R. solanacearum* escaped from the vascular ring as early as 5 dpi and heavily colonized the pith and cortical tissues of Marmande by 9 dpi (Fig. 7). Moreover, the amount of bacteria outside the vascular tissues was directly correlated with the extent of vascular ring colonization (Fig. 7B). This contrasted with the ability of H7996 shoots to impede the escape of the pathogen from the vascular ring (Figs 6 and 7). These results indicate that the capacity of *R. solanacearum* to radially invade the pith and cortex tissues is dependent on the degree of susceptibility of the plant and that invasion occurs as a consequence of increased colonization.

## Discussion

**Fig. 8.**
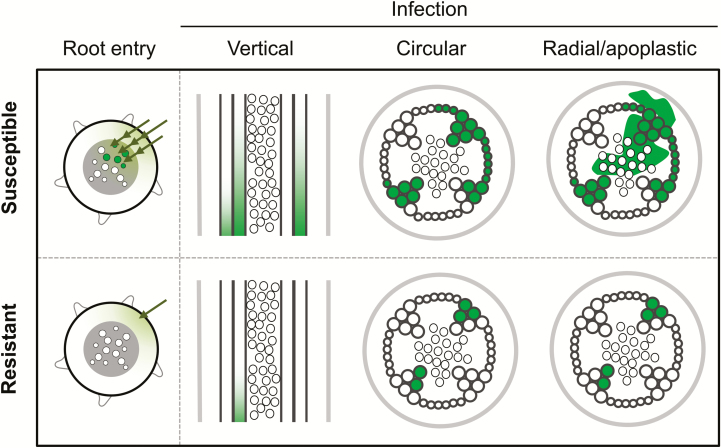
Model of the tomato–*R. solanacearum* pathosystem in susceptible and resistant germplasm. Schematic representation of the colonization movements of *R. solanacearum* (shown in green) in susceptible and resistant tomato tissues.

In this work we propose a model that relates the spatio-temporal dynamics of *R. solanacearum* invasion and proliferation in tomato plants to disease development and shows how quantitative resistance affects these parameters (Fig. 8). Systematic analysis of the progression of bacterial colonization inside the plant reveals four clear limitations of growth in resistant tomato tissues that hamper disease progression: colonization of the root, invasion of the stem vascular bundle, vertical invasion up the vessels, and invasion of the pith/cortex. The basically binary outcome of death-by-permanent-wilting caused by *R. solanacearum* in tomato seems to require the bacterium to surmount each of these physio-anatomical plant barriers, which are quantitatively determined by host resistance. We will discuss each of these four important ’bottlenecks’ that can determine either host resistance or successful colonization of the plant by the bacterium.

### Restriction of root colonization

We analyzed the interaction of *R. solanacearum* with tomato using two main variables: susceptible versus resistant varieties and soil drenching versus pin-inoculation. Soil-drenching inoculations clearly reproduced the progression of disease and the resistance observed in controlled-environment studies with comparable conditions and plant ages for the different varieties investigated (Supplementary Fig. S2; Wang *et al.*, 1998; McGarvey, Denny and Schell, 1999; Nakaho *et al.* 2004; Rivard and Louws, 2008). Root pin-inoculation of plantlets grown *in vitro* showed similar results (Fig. 1A), but bacterial concentrations reached higher numbers in the tissues of pin-inoculated resistant varieties compared with soil-drenched plants of the same resistant varieties, while the susceptible variety was highly colonized by both methods of inoculation (Figs. 1 and 2, Supplementary Fig. S1). The differences in the inoculation methods imply that resistant varieties have the ability to restrict invasion of the root—a step that is overcome when pin-inoculation of the roots is used-. Our findings are in agreement with the limited bacterial growth in the taproot of H7996 observed when roots were not wounded before inoculation (McGarvey *et al.*, 1999). Additionally, *in vitro*-grafted pin-inoculated plants displayed slightly delayed colonization compared with non-grafted plants (3 dpi in Fig. 1A versus 5 dpi in Fig. 2). This difference might be linked to the developmental stage. Since older plants (in this case the grafted ones) are more developed, their cell walls might be reinforced, thus partly hindering *R. solanacearum* invasion. Finally, the pin-inoculated resistant plants that are highly colonized likely mimic the situation encountered in nature when environmental conditions are highly favorable to the pathogen. Indeed, it has been shown that even the most highly resistant varieties that are available cannot completely prevent root and stem colonization by *R. solanacearum* in greenhouse conditions (Nakaho, 1997*a*, *b*; Nakaho *et al.*, 2004).

### Restriction of vertical movement up the stem

The fact that *R. solanacearum* can colonize the stems of many resistant tomato plants when soil-drenching is used as the method of inoculation indicates that additional mechanisms of resistance must be in place in the aerial tissues to prevent wilting. Previous studies have demonstrated that bacterial counts in the stems of resistant tomato plants were always lower than those in susceptible varieties and that this was due to a limitation of pathogen movement from the primary xylem to other xylem tissues (Nakaho *et al.*, 2004). In this work, we have analyzed the vertical dimension of bacterial spread and demonstrated that resistant tissues limit movement up the xylem vessels (Fig. 3). Double-grafting experiments, in which a small portion of resistant stem was introduced into an otherwise susceptible adult plant or *vice versa*, ruled out any effect of grafting *per se* on bacterial movement inside the xylem and suggested that resistance to bacterial wilt could be due to non-diffusible xylem structures/compounds originating from the stem, as has been described for other bacterial vascular diseases (Chatterjee *et al.*, 2008).

The nature of the plant components or structures hindering root-to-shoot vertical bacterial movement is still unknown, although reports have described the presence of tyloses (evaginations of the adjacent parenchyma cells into the xylem lumen) and gums that seemed to limit bacterial spread in the xylem of bacterial wilt-resistant Caraïbo tomato plants (Grimault *et al.*, 1994*b*). Obstruction of xylem vessels with gums and tyloses is a common plant response to restrict systemic infection by vascular pathogens (VanderMolen *et al.*, 1987; Grimault *et al.*, 1994; Clérivet *et al.*, 2000; Sun *et al.*, 2013). For instance, vascular gelation is considered an essential part of *Fusarium* wilt resistance in carnation (Baayen and Elgersma, 1985). Tyloses have been similarly proposed to restrict pathogen movement in tomato cultivars resistant to the vascular pathogens *Fusarium oxysporum, Verticillium abo-atrum,* and *R. solanacearum* (Hutson and Smith, 1980; VanderMolen *et al.*, 1987; Grimault *et al.*, 1994*b*). Although Grimault *et al.* (1994*b*) correlated the presence of tyloses in Caraïbo to the limitation of *R. solanacearum* spread, in another resistant cultivar (LS-89) the formation of these structures was neither induced by the pathogen nor seemed to affect bacterial colonization (Nakaho, 1997*a*). Similarly, tyloses formed in grapevines in response to *Xylella fastidiosa* infection were found to be more abundant in susceptible cultivars and did not affect the vertical movement of the pathogen (Sun *et al.*, 2013). These observations suggest that the role of tyloses in vascular pathogen restriction may be cultivar- or species-specific and/or depend on the lignification status of the plant host. The results presented here and our recent finding that *R. solanacearum*-tolerant potato lines also induced the development of tyloses upon infection (Ferreira *et al.*, 2017) seem to indicate that these structures are important components of bacterial wilt resistance in solanaceous plants.

### Restriction of vascular bundle invasion and the bacterial density threshold

Restriction of *R. solanacearum* colonization in the stems of H7996 is also achieved by limiting the horizontal movement of the pathogen from vessel to vessel (referred to hereafter as circular movement). Confinement of *R. solanacearum* to primary xylem vessels has been observed in the stems and roots of different resistant tomato cultivars compared with susceptible ones (Nakaho, 1997*a*; Nakaho *et al.*, 2004; Caldwell *et al.*, 2017). A similar correlation between *R. solanacearum* movement between stem vessels, bacterial growth, and the level of susceptibility has been observed in potato (Cruz *et al.*, 2014; Ferreira *et al.*, 2017). Similarly, *X. fastidiosa* has been shown to invade 10 times fewer stem vessels and exhibit lower population densities in resistant grapevine cultivars (Chatterjee *et al.*, 2008). These results indicate that limitation of circular movement in the xylem ring is a conserved mechanism for resistance against vascular bacterial pathogens. Restriction of *R. solanacearum* to the primary xylem vessels could explain why resistant tomato plants often remain asymptomatic. If a blockage occurs in the primary xylem, which is largely non-functional after the secondary xylem has been produced (Esau, 1977), flow conduction could occur undisturbed through the uninfected secondary xylem.

In susceptible plants, *R. solanacearum* can move horizontally through the xylem ring by directly degrading the cell walls of primary xylem vessels or pit membranes in the secondary xylem vessels (Wallis and Truter, 1978; Grimault *et al.*, 1994*b*; Vasse *et al.*, 1995; Nakaho *et al.*, 2000). To counter such circular movement, plants have evolved structural defenses that are induced upon attack by vascular pathogens, involving the deposition of various coating materials to reinforce the walls of xylem vessels, pit membranes, and surrounding parenchyma cells. Vascular coatings are thicker in resistant tomato cultivars infected with *R. solanacearum*, and this may be the cause of the observed limitation of bacterial movement between xylem tissues (Nakaho *et al.*, 2000, 2004). The detailed description of the process we present here will be crucial to decipher the genetic determinants and the composition of these vascular coatings, which remain unknown.

Restriction of circular movement in the stem is a very efficient confinement strategy, since it is still acting when high loads of bacteria are forced into the stem through root inoculations using H7996 scions grafted on to Marmande rootstocks (Fig. 6). However, there seems to be an upper limit of bacterial inoculum above which this restriction is no longer effective (see plant 4 in the lower panel of Fig. 6C). This finding is in agreement with previous reports showing that delivery of a high *R. solanacearum* inoculum (10^9^ CFU ml^–1^) directly into tomato stems overcomes resistance (Nakaho, 1997*b*). This idea relates to the concept of a density threshold in the interaction between tomato and *R. solanacearum*. Earlier observations established the onset of bacterial wilt symptoms at a bacterial density in the stem of between 10^6^ and 10^8^ CFU g^–1^ fresh tissue (Grimault and Prior, 1994; Nakaho, 1997*a*; Huang and Allen, 2000; Nakaho *et al.*, 2004). We have characterized this threshold systematically by assessing bacterial densities throughout the plant in large populations of grafted tomato plants with varying resistance. We conclude that, in both resistant and susceptible varieties, symptoms invariably appear when bacterial populations in the hypocotyl exceed a threshold of 10^7^ CFU g^–1^ tissue (Fig. 5, Supplementary Fig. S7). Plating dilutions of homogenized tissues to establish bacterial counts is labor intensive, but we show that light emission from tissues inoculated with a luminescent strain is a useful alternative measure of bacterial counts (correlation coefficient r^2^=0.96). Since bacterial density and distribution are predictive of the degree of disease resistance, we have started to use luminescent strains to screen potato germplasm for resistance to bacterial wilt as a way of aiding the breeding process (Cruz *et al.*, 2014; Ferreira *et al.*, 2017).

### Restriction of radial movement out of the xylem into the pith and cortex

Our work has also revealed a fourth limitation restricting bacterial spread in the tissues of resistant tomato varieties: restriction of radial movement of *R. solanacearum* out of the xylem into the adjacent parenchyma cells in the pith and cortex (Figs 6 and 7). These metabolically active cells are in close contact with the xylem vessels through the pits and are thought to be pivotal for the induction of plant defenses against pathogens within the xylem, although very little is known about the mechanisms regulating this response.

Earlier studies detected widespread *R. solanacearum* colonization of stem parenchyma cells in susceptible tomato varieties at late stages of infection, when plants showed extensive wilting (Nakaho, 1997*a*; Nakaho *et al.*, 2000). These cells appeared to be filled with bacteria and displayed necrosis and signs of degeneration. In contrast, in resistant tomato varieties, necrotic parenchyma cells containing bacteria were observed only occasionally (Nakaho *et al.*, 2000). Our data confirm these observations and additionally show that parenchyma cell invasion starts at early times in susceptible plants (5 dpi in our experimental setup) and spreads massively through the pith at later time points (8–9 dpi, Fig. 7A). In contrast, colonization remains limited to xylem vessels in resistant tomato (Fig. 6).

As was the case for the circular bacterial movements described above, radial restriction of movement out of the xylem in resistant varieties could be partially overridden by grafting to susceptible rootstocks that enabled high bacterial densities to access resistant tissues, as can be seen in some of the images in Fig. 6C. This observation is in agreement with a previous report showing that when high bacterial inocula (10^9^ CFU ml^–1^) were used, *R. solanacearum* could also be detected in the parenchyma cells of resistant tomato (Nakaho, 1997*b*). Thus, restriction of radial bacterial movement is no longer effective when the bacterial density exceeds a certain threshold.

Structural changes in cell walls and pit membranes in response to *R. solanacearum* infection are more conspicuous in resistant tomato (Nakaho *et al.*, 2000). Thus, bacteria may be prevented from escaping the xylem in resistant tomato by a combination of inducible structural defense mechanisms that may appear later and/or with less intensity in susceptible lines, rendering them ineffective to restrict colonization. Interestingly, slightly decreased invasion was also observed in the susceptible hypocotyls of the Marmande–H7996 grafting combination (Fig. 7). This finding could be explained by a cross-talk between the scion and rootstock. This interaction could trigger the expression of putative defense-related genes or genes that reinforce plant cell wall structures in the susceptible rootstock. Alternatively, defense-related or structure-remodeling proteins might be secreted by the resistant scion and reinforce nearby tissues (in this case the susceptible hypocotyl). These two explanations seem plausible given the existing vascular connectivity between the grafted tissues. Indeed, transcriptional reprogramming occurs even in rootstocks and scions of the tomato/potato heterografting system (Zhang *et al.*, 2019). Additionally, peroxidases and other cell wall remodeling enzymes, such as glycosyl hydrolases, are secreted into the xylem by the resistant line H7996 upon *R. solanacearum* infection (M. Planas-Marquès, F. Kaschani, M. Kaiser, R. A. L. van der Hoorn, M. Valls, N. Sánchez-Coll, unpublished results). Hence, increased lignification and cell wall reinforcement could also take place in neighboring susceptible tissues in grafted plants.

### An integrated model for tomato resistance to bacterial wilt

As we have discussed, in the past three decades, various laboratories have aimed to understand how resistant tomato varieties restrict *R. solanacearum* colonization and remain asymptomatic despite having relatively high bacterial loads. The fact that the defenses are not limited to a particular plant site, as the bacterium has to traverse different tissues to reach the xylem, has complicated this work. A question that arises is whether the xylem is the final destination of *R. solanacearum*.

Here, we have defined the barriers encountered by *R. solanacearum* as it progresses from the soil into the xylem and have found that, after systematic spread through the xylem, the final destination of the bacterium may be extensive invasion of the stem apoplast. It has already been suggested that vascular bacteria use plant cell wall degradation products as carbon and energy sources (Chatterjee *et al.*, 2008; Genin and Denny, 2012). It is tempting to speculate that *R. solanacearum* has evolved not only to colonize the xylem but also to escape from it to obtain richer sources of nutrition from metabolically active parenchyma cells, facilitating the death and decay of infected plants and thus allowing the pathogen to spread in the soil and move into the next host.

In conclusion, here we clearly define four ‘bottlenecks’ to bacterial colonization in tomato and demonstrate that the degree of resistance of a given variety correlates with its capacity to restrict bacterial movement at these locations. The ability to restrict bacterial movement at all four anatomical points makes H7996 the most resistant tomato line, consistent with the polygenic nature of its resistance (Wang *et al.*, 2013), which has made introgression breeding extremely difficult (Scott *et al.*, 2005; Hanson *et al.*, 2016). We believe that this integrative study will serve as a first step towards the characterization of the genetic and molecular determinants that govern resistance at each stage of *R. solanacearum* invasion.

## Supplementary data

Supplementary data are available at *JXB* online.

Fig. S1. Measurement of bacterial root colonization in tomato plants.

Fig. S2. Symptom development over time in grafted tomato plants.

Fig. S3. Correlation between luminescence and bacterial counts.

Fig. S4. *R. solanacearum* vertical movement in tomato shoots as seen by different intensities of exposure.

Fig. S5. *R. solanacearum* vertical movement in the shoots of Marmande and Shield grafted plants.

Fig. S6. Disease evolution over time in double-grafted plants.

Fig. S7. *R. solanacearum* bacterial density assessed over the height of grafted tomato plants.

Fig. S8. Circular and radial invasion of *R. solanacearum* in susceptible and resistant tomato shoots.

Fig. S9. Invasion of *R. solanacearum* in susceptible and resistant pin-inoculated tomato shoots.

erz562_suppl_Supplementary_Figures_S1-S9
