# Supplementary material for: Four bottlenecks restrict colonization and invasion by the pathogen Ralstonia solanacearum in resistant tomato
Source: J Exp Bot. 2019 Dec 24;71(6):2157–71. doi: 10.1093/jxb/erz562 (PMC7242079; doi:10.1093/jxb/erz562)
Supplement: erz562_suppl_Supplementary_Figures_S1-S9 [file erz562_suppl_supplementary_figures_s1-s9.pdf]

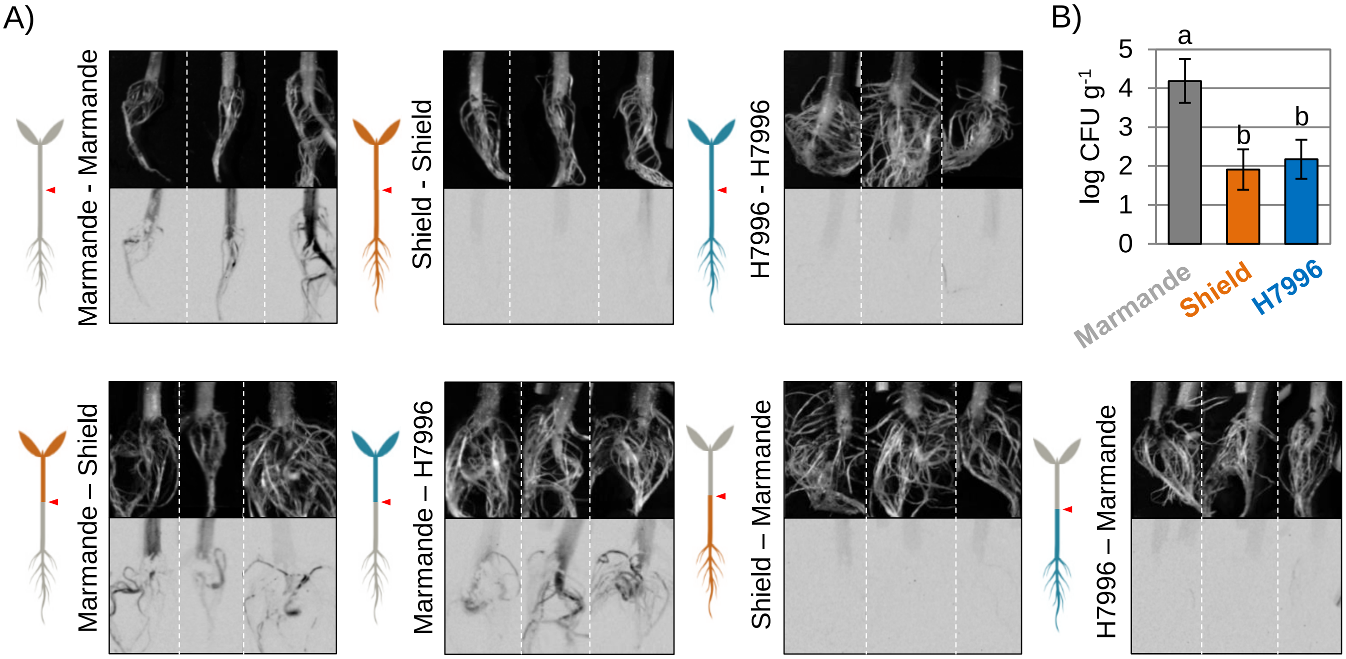

Fig. S1. Measurement of bacterial root colonization in tomato plants. Five-to-six week-old grafted tomato plants were soil-inoculated with the luminescent *R. solanacearum* strain and root luminescence was evaluated over time. A) White light (top) and luminescence (bottom) photographs taken at 6 dpi from representative roots from grafted Marmande (susceptible), Shield (moderately resistant to bacterial wilt), and H7996 (highly resistant to bacterial wilt). The plant outline is due to background light from photosynthetic tissues, while luminescence is detected as darker areas. Saturation level was never reached. B) Mean bacterial densities (log CFU g^{-1}) measured at 3 dpi from 0.5 cm sections of the taproot for each variety, inoculated as explained above. Error bars indicate standard error. n=24 plants per variety. Letters above bars indicate significant statistical difference by Fisher's LSD (α=0.05).

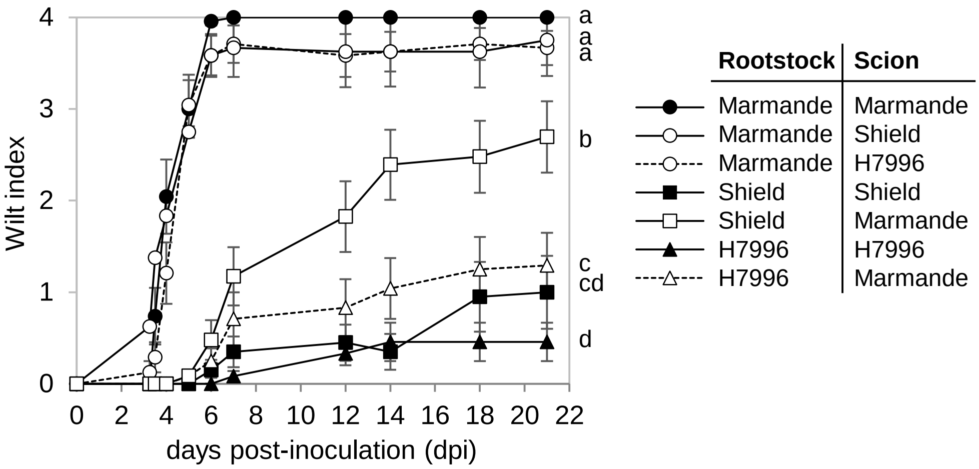

Fig. S2. Symptom development over time in grafted tomato plants. Five-to-six week-old grafted tomato plants were soil-inoculated with the luminescent *R. solanacearum* strain and wilting symptoms were scored over time. Error bars indicate standard errors. n=20-24 plants per grafting treatment. Letters indicate significant statistical difference at 21 dpi by Fisher's LSD (α=0.05).

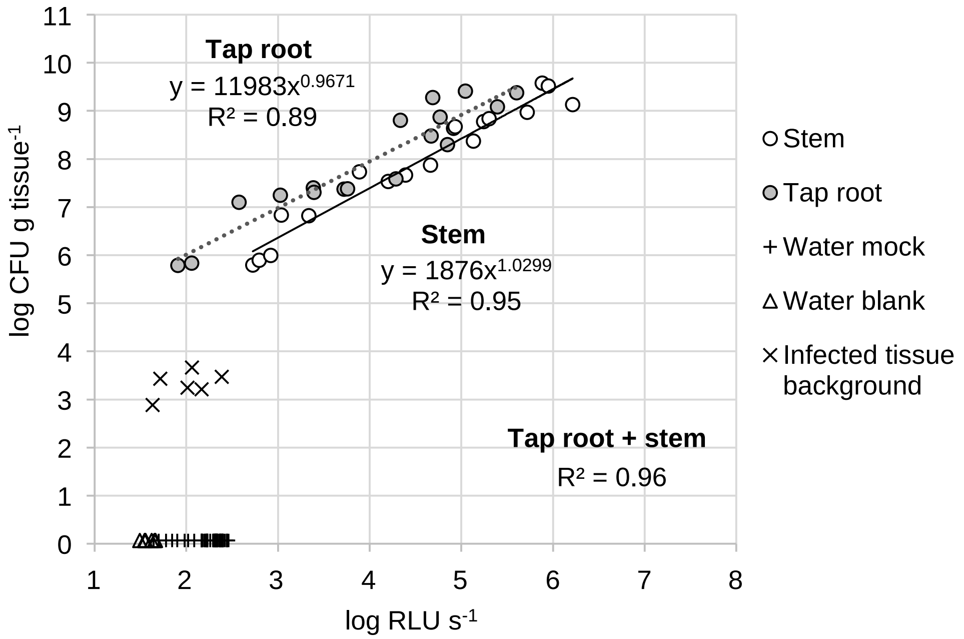

Fig. S3. Correlation between luminescence and bacterial counts. Fragments (0.5 cm) of infected tissues were placed in 200 µl sterile water and luminescence was measured using a luminometer. Dilution plating was carried out on the same samples in order to count Colony Forming Units (CFU). Luminescence values (RLU s^{-1}) were plotted against their CFU counter-part. Two trend lines (for taproot and for stem sections) were plotted to obtain the equations used in posterior calculations. "Water mock" samples represent 0.5 cm stem fragments of mock-inoculated plants (RLU s^{-1} tissue background), while "Water blanks" do not contain any tissue fragment. Finally, "Infected tissue background" refers to samples that showed background-like luminescence signal but were colonized by *R. solanacearum* (i. e. the detection threshold).

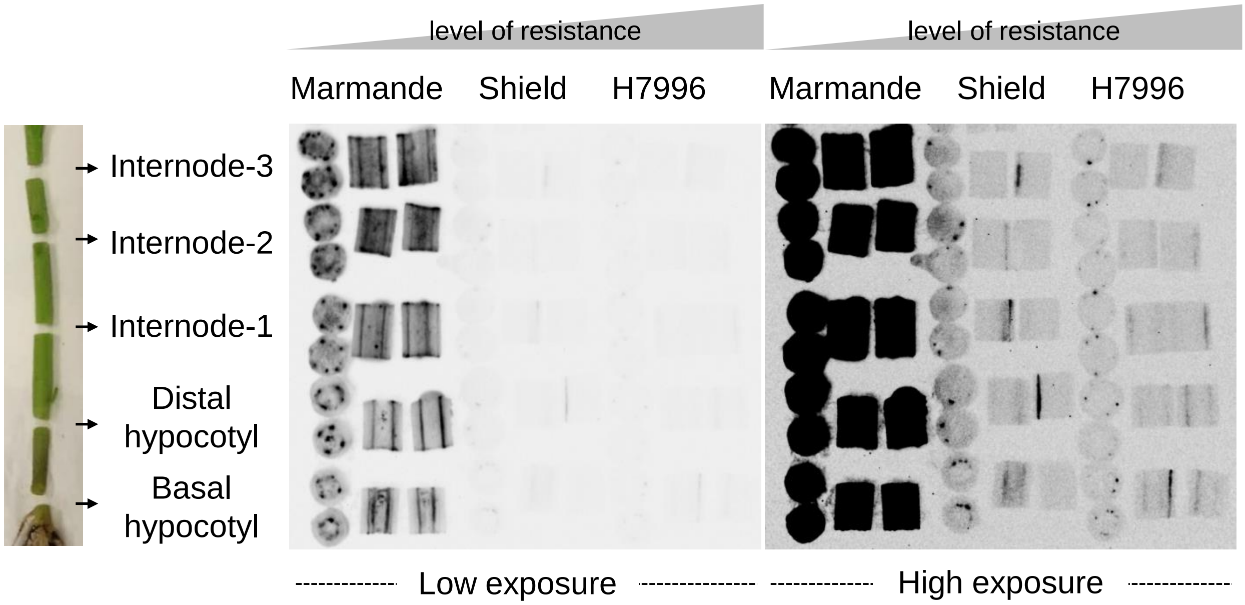

Fig. S4. *R. solanacearum* vertical movement in tomato shoots as seen by different intensities of exposure. Four-to-five week-old tomato plants of non-grafted susceptible Marmande, the moderately resistant Shield variety, and the highly resistant H7996 were grown in pots and soil inoculated with the luminescent *R. solanacearum.* Shoot sections were obtained at 6 dpi. For each shoot fragment, top and bottom slices were obtained and the fragment was vertically bisected before exposure to a live imager. This figure shows the luminescence photograph from Figure 3A using the two different exposure settings on the Image Lab software (Bio-Rad): 'High'/'Low'/'Gamma' values of 10000/60/1 and 1300/60/2 for the low and high exposures, respectively.

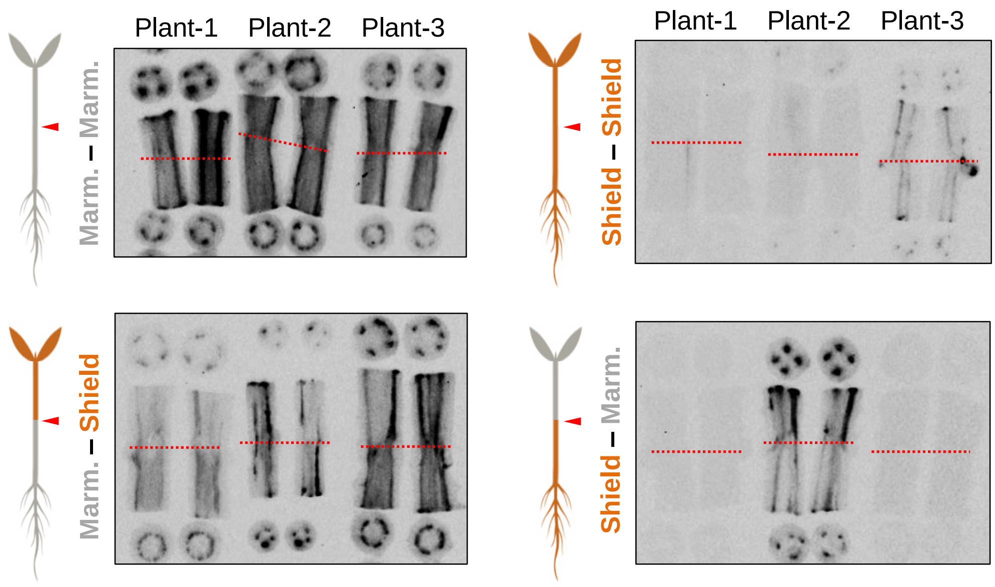

Fig. S5. *R. solanacearum* vertical movement in the shoots of Marmande and Shield grafted plants. Five-to-six week-old reciprocally grafted Marmande and Shield plants were soil inoculated with the luminescent *R. solanacearum.* Shoot sections were obtained at 7 dpi and photographed in a live imager. Photographs represent each bisected fragment and its top and bottom slices exposed. Sections were obtained above and below the graft junction. The arrowheads and dotted lines indicate the position of the graft union.

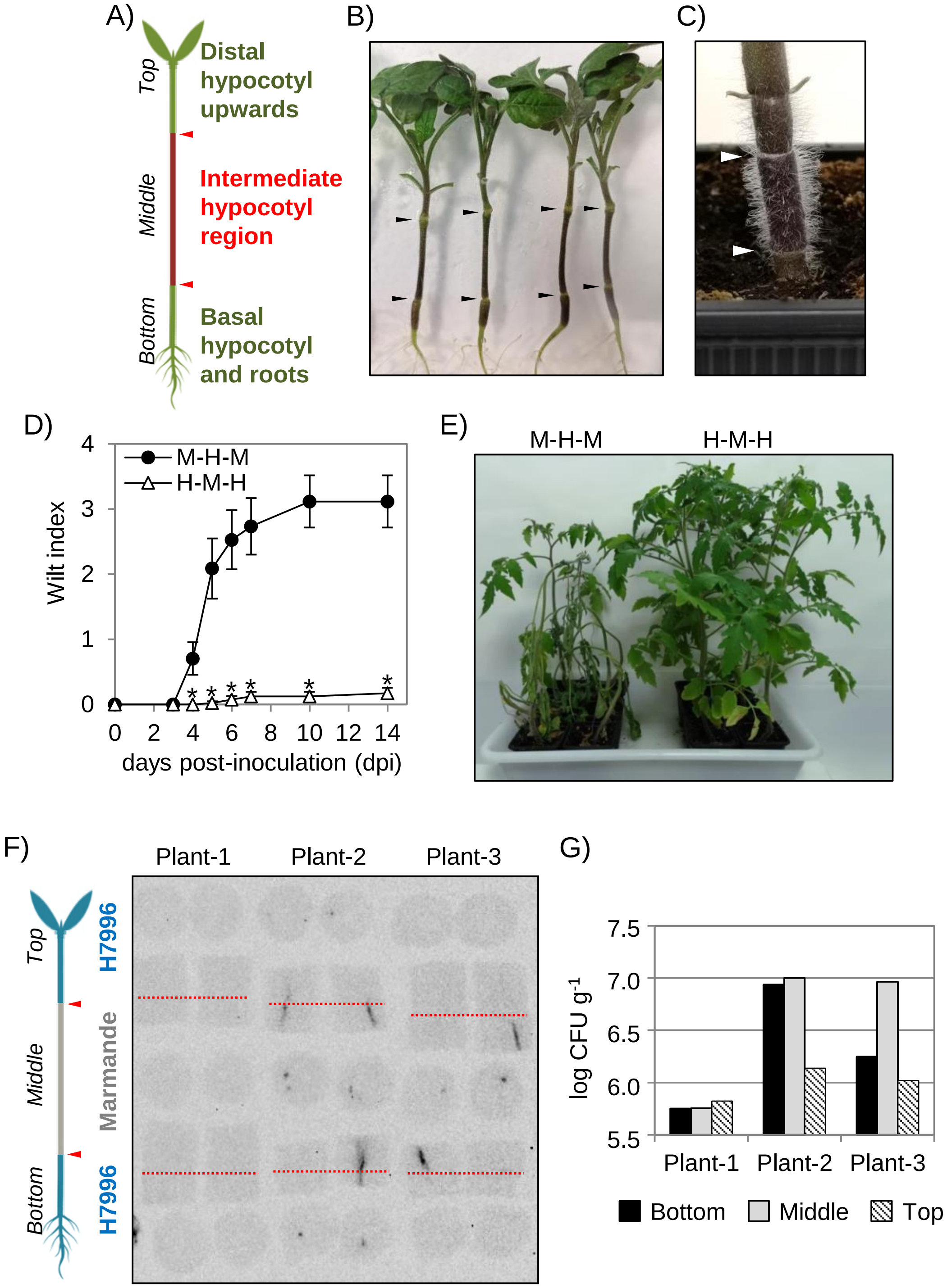

Fig. S6. Disease evolution over time in double-grafted plants. A) Schematic representation of a double-grafted plant. B) Eight day-old tomato seedlings were grafted in vitro and after 8 days of recovery they were transferred to pots. Arrowheads point the grafting unions. C) Close-up picture of the perfectly healed grafting unions (arrowheads) of double-grafted plants grown in pots for 3-4 weeks. D) Five-to-six week-old double-grafted plants were inoculated with the luminescent *R. solanacearum* strain and wilting symptoms were scored over time. Error bars indicate standard error. Asterisks indicate significant statistical difference by t-test (α=0.01). M-H-M, Bottom:Marmande – Middle:H7996 – Top:Marmande (n=17). H-M-H, Bottom:H7996 – Middle:Marmande – Top:H7996 (n=20). E) Wilting symptoms at 10 dpi. On the left, 6 M-H-M representative plants. On the right, 6 representatives of the reciprocal grafting. F) Shoot sections from the hypocotyl of 10 dpi H-M-H plants were photographed in a live imager. The two graft unions are indicated by arrowheads and dotted lines. G) Bacterial loads were quantified in the shoots of the plants shown on (F) using the luminescence-CFU correlation.

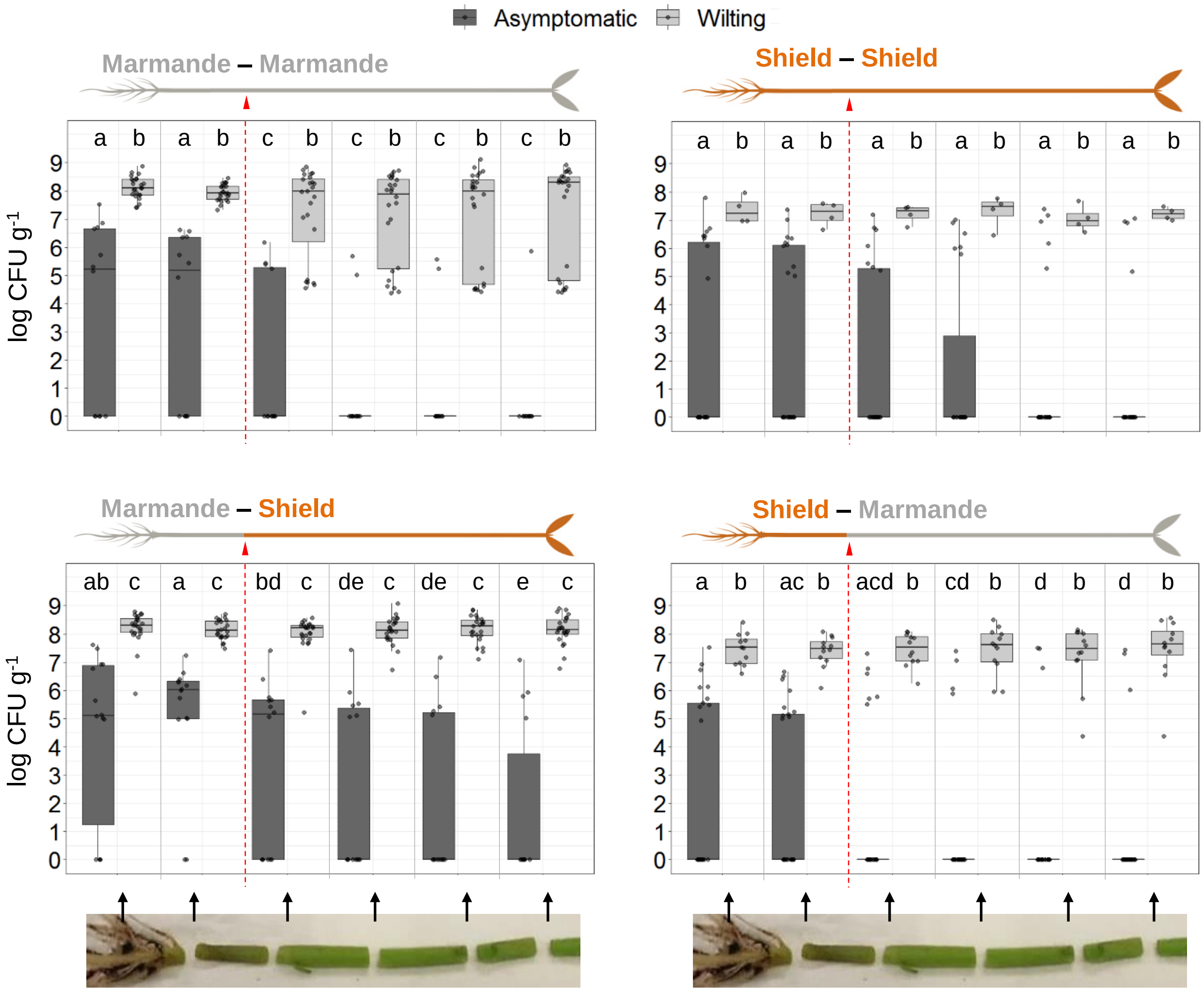

Fig. S7. *R. solanacearum* bacterial density assessed over the height of grafted tomato plants. Bacterial concentrations at different heights in the tissues of wilting (light grey) and asymptomatic (dark grey) grafted plants. Luminescence was measured with a luminometre in 0.5 cm sections from at least 27 inoculated plants per grafting combination. Bacterial counts were calculated from luminescence and are expressed as log CFU g tissue-1. Each dot represents one plant. Values between 0 and 4 lie below the threshold for luminescence detection (see Supplementary Figure S3) and are here considered as zeros. From left to right, sections correspond to: taproot, basal hypocotyl, distal hypocotyl, internodes 1, 2 and 3. The dashed red line highlights the location of the grafting union. Letters above each boxplot indicate significant statistical difference by Fisher's LSD (α=0.05). Within each boxplot, the whiskers extend from the hinges to the largest (upper whisker) or smallest (lower whisker) value no further than 1.5 * IQR from the hinge (where IQR is the inter-quartile range, or distance between the first and third quartiles). Dots beyond the end of the whiskers are outliers. The band inside each box indicates the median. The Marmande-Marmande panel is the same as the one presented in Fig 5.

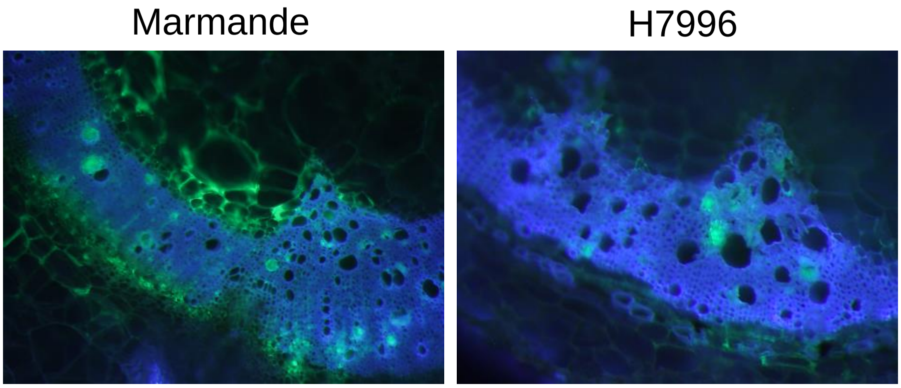

Fig. S8. Circular and radial invasion of *R. solanacearum* in susceptible and resistant tomato shoots. Grafted plants containing H7996 scions on Marmande rootstocks were inoculated with the fluorescent strain and transversal sections were photographed in a fluorescence stereomicroscope. Sections shown here are from lowly colonized Marmande (left panel) and H7996 (right panel) shoots obtained 9 days post-inoculation (dpi). The H7996 section corresponds to a magnification of Plant-2 from Fig. 6C. Green areas indicate *R. solanacearum* strain; blue indicates lignin autofluorescence.

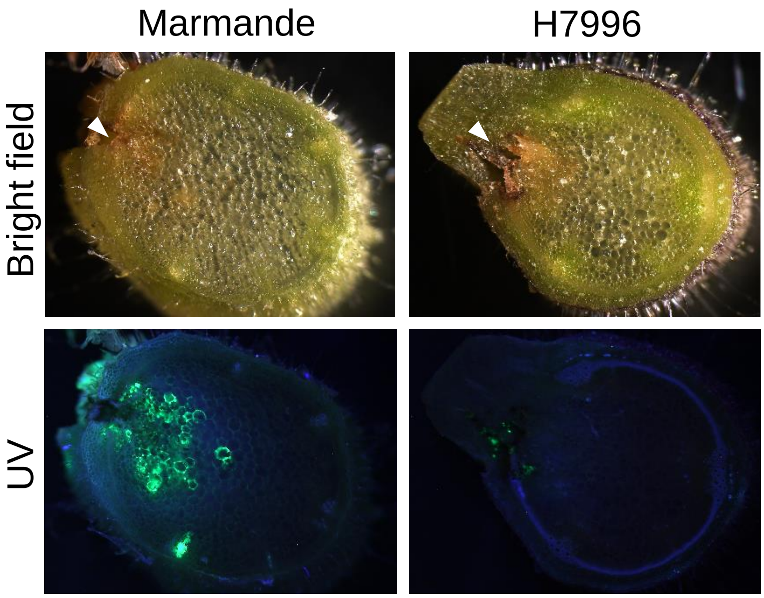

Fig. S9. Invasion of *R. solanacearum* in susceptible and resistant pin-inoculated tomato shoots. Four-to-five week old tomato plants were pin-inoculated at the second internode with a fluorescent *R. solanacearum* strain. The plants were sectioned 5 days post-infection at the inoculation point and sections were visualized in a stereomicroscope using a UV filter. Green areas indicate *R. solanacearum* strain; blue indicates lignin autofluorescence. The arrowhead in the bright field photograph indicates the inoculation point.
